# A novel 11β-hydroxysteroid dehydrogenase type1 inhibitor CNX-010-49 improves hyperglycemia, lipid profile and reduces body weight in diet induced obese C57B6/J mice with a potential to provide cardio protective benefits

**DOI:** 10.1186/2050-6511-15-43

**Published:** 2014-08-07

**Authors:** Tharappel M Anil, Anilkumar Dandu, KrishnaReddy Harsha, Jaideep Singh, Nitya Shree, Venkatesh Satish Kumar, Mudigere N Lakshmi, Venkategowda Sunil, Chandrashekaran Harish, Gundalmandikal V Balamurali, Baisani S Naveen Kumar, Aralakuppe S Gopala, Shivakumar Pratibha, ManojKumar Sadasivuni, Mammen O Anup, Yoganand Moolemath, Marikunte V Venkataranganna, Madanahalli R Jagannath, Baggavalli P Somesh

**Affiliations:** 1 Connexios Life Sciences Pvt Ltd, Bangalore, India

**Keywords:** 11β-HSD1, CNX-010-49, Glucose, Insulin sensitivity, Triglycerides, Adipogenesis and Body weight, Type 2 Diabetes, Cardiovascular risks

## Abstract

**Background:**

11ß–hydroxysteroid dehydrogenase type1 (11β-HSD1) converts inactive glucocorticoids to active glucocorticoids which, in excess, leads to development of the various risk factors of the metabolic syndrome. Recent studies clearly suggest that both increased expression and activity of 11β-HSD1 in metabolically active tissues such as liver, muscle and adipose are implicated in tissue specific dysregulation which collectively contribute to the whole body pathology seen in metabolic syndrome. In the present study we have evaluated CNX-010-49, a highly potent, selective and ‘pan tissue’ acting 11β-HSD1 inhibitor, for its potential to modulate multiple risk factors of the metabolic syndrome.

**Methods:**

Male C57B6/J mice on high fat diet (DIO mice) were orally dosed with CNX-010-49 (30 mg/kg twice daily; n = 8) or vehicle for 10 weeks. Fasting glucose, triglycerides, glycerol, free fatty acids, body weight and feed intake were measured at selected time points. At the end of the treatment an OGTT and subsequently organ histology was performed. *In vitro*, CNX-010-49 was evaluated in 3T3-L1 preadipocytes to assess impact on adipocytes differentiation, hypertrophy and lipolysis whereas in fully differentiated C2C12 cells and in primary mouse hepatocytes to assess the impact on glucose metabolism and hepatic glucose output respectively.

**Results:**

CNX-010-49 a highly potent and selective pan tissue acting 11β-HSD1 inhibitor (EC_50_ = 6 nM) significantly inhibits glucocorticoids and isoproterenol mediated lipolysis in mature 3T3-L1 adipocytes, improves muscle glucose oxidation, reduces proteolysis and enhances mitochondrial biogenesis. Also a significant inhibition of gluconeogenesis in primary mouse hepatocytes was observed. The treatment with CNX-010-49 resulted in a significant decrease in fasting glucose, improved insulin sensitivity and glucose tolerance. Treatment also resulted in a significant decrease in serum triglycerides levels and a complete inhibition of body weight gain without affecting feed consumption. A significant reduction in the serum biomarkers like Plasminogen activator inhibitor-1 (PAI-1), interleukin 6 (IL-6) and Fetuin-A with CNX-010-49 treatment was observed indicating a potential to modulate processes implicated in cardiovascular benefits.

**Conclusions:**

These results indicate that inhibition of 11β-HSD1 with CNX-010-49 can give a potential benefit in the management of metabolic dysregulations that are seen in type 2 diabetes.

## Background

Glucocorticoids (GCs) play a critical role in multiple metabolic processes, including glucose homeostasis, insulin sensitivity and lipid metabolism. Also it is well established that elevated glucocorticoid levels is linked with increased cardiovascular events [1]. Metabolic syndrome and Cushing’s syndrome have some similar phenotypes that include hyperglycemia, visceral obesity, dyslipidemia and insulin resistance [2].

There are two isoforms of 11β-Hydroxysteroid dehydrogenase. 11β-HSD1 which converts the inactive 11β-keto form (cortisone in humans and 11-dehydrocorticosterone in rodents) to the active 11β-hydroxylated GCs (cortisol in human and corticosterone in mice) [3]. It is expressed primarily in GC target tissues such as liver, skeletal muscle, adipose tissue and in the central nervous system where it amplifies local GC action [4]. 11βHSD2 is highly expressed in classical aldosterone-selective target tissues, such as kidney [5] and prevents the inappropriate activation of mineralocorticoid receptors by cortisol. In conditions of excess NADPH, as seen in metabolic overload; 11β-HSD1 functions as a reductase and generates active cortisol while in situations of low levels of NADPH it functions as a dehydrogenase and generates inactive cortisone [6]. In humans, *in vivo* dehydrogenase activity of 11β-HSD1 has been demonstrated [7].

Animals administered GCs show similar dysregulation in phenotype as seen in metabolic syndrome or T2DM where they observed increased fasting hyperglycemia, hyperinsulinemia and impaired β-cell response to oral glucose challenge. These animals have shown hepatic steatosis and increased ectopic lipid accumulation in muscle [8]. In adipocytes GCs increase lipolysis and hypertrophy in mature adipocytes [9] whereas in muscle GCs can increase proteolysis and insulin resistance. In another study, infusion of GCs has also shown in addition to the above observations hyperleptinemia, hypertriglyceridemia, significant decrease in uncoupling protein (UCP)-1 and UCP-3 expression [10]. The loss of UCP1 expression is shown to be attendant with decrease in non-shivering thermogenesis [11,12].

In metabolic syndrome, the pathology is due to enhanced tissue level glucocorticoids [13]. Targeted disruption of the 11β-HSD1 leads to improvement in glucose tolerance, improved lipid profile along with decreased gluconeogenic response [14]. Mice over expressing adipose 11β-HSD1 develop visceral obesity which is further exacerbated by feeding high fat diet. These mice later developed all the phenotypes of metabolic syndrome including hypertension [15,16].

11β-HSD1 expression and activity are significantly increased in both skeletal muscle and fat tissue from obese type 2 diabetes (T2DM) patients and also in rodent models of disease suggesting a role for local glucocorticoids re-amplification in the development of obesity and the metabolic syndrome [13,17-20]. Also increased 11β-HSD1 expression and activation in liver and adipose has demonstrated a clear link between its roles to T2DM as seen with glucose intolerance, increased insulin resistance, increased adiposity and body weight gain [21,22]. The concomitant increase in glucocorticoids in adipose leads to decreased adiponectin levels, increased TNFα and fasting glucose whereas hepatic overexpression increased insulinemia, LDL cholesterol and serum glucose levels. One study established the association impaired insulin signaling and 11β-HSD1 expression/activity in skeletal muscle where dexamethasone treated myotubes showed reduced IRS1 expression, increased Ser307 phosphorylation of IRS1 and reduced downstream pSer473 Akt/PKB [23].

Pharmacological inhibition of 11β-HSD1 in different rodent models has demonstrated an improvement in glucose tolerance, insulin sensitivity as well as reduced body weight gain [24-30]. Also 11β-HSD1 inhibition reduced serum triglycerides, cholesterol and frees fatty acids levels. Importantly, inhibition of 11β-HSD1 reduces plaque progression and aortic cholesterol accumulation murine model of atherosclerosis.

To date a few small molecule inhibitors of 11β-HSD1 have entered clinical studies. INCB13739 displayed statistically significant reductions in HbA1c and glucose in T2DM patients where metformin monotherapy was inadequate [31]. MK-0916 decreased both blood pressure and body weight with a trend to reduce waist circumference and had no significant effect on blood glucose [32].

So far none of these interventions provided significant overall protection from metabolic syndrome. One can attribute this lack efficacy of 11β-HSD1 inhibitors may be due to potential reversibility of the 11β-HSD1 enzymatic reaction. Selection of 11β-HSD1 inhibitors which inhibit reductase activity than dehydrogenase activity and getting maximum inhibition in skeletal muscle apart from adipose and liver is very important. So there is still a need for intervention that alters 11β-HSD1 enzyme activity and thereby provides a significant benefit in the management of metabolic syndrome.

Our understanding of 11β-HSD1 biology linking to metabolic syndrome suggests that inhibition of 11β-HSD1 with highly potent compound in all the metabolically active tissues like adipose, skeletal muscle and liver, will provide a complete benefit in controlling the disease. Also we screened and selected the compounds that have shown more inhibition of reductase activity than dehydrogenase activity along with good tissue distribution and inhibition in the above mentioned tissues. In this study, we have evaluated CNX-010-49 (MW = 408.5), a highly potent and selective 11β-HSD1 inhibitor for its potential to control multiple facets of metabolic syndrome.

## Methods

### Cell culture and treatment

C2C12 cells (Mouse myoblasts, ATCC) were maintained in 24-well plates (3X10^4^ cells/well) at 37°C in DMEM (Dulbecco’s modified Eagle’s medium) containing 25 mM glucose and 10% FBS. When cells reached confluence, the media was supplemented with 25 mM Glucose and 2% FBS for myotubes formation. After 4 days of differentiation and myotubes formation, cells were treated with media containing GPCI (25 mM glucose, 500 μM palmitate, 500 nM cortisone, 10 ng/ml of TNFα, IL-1β & IFγ) with or without 1 μM of CNX-010-49 for 18 h. Post 18 h, cells were frozen in TRIZOL (Sigma, USA) for further analysis.

3T3-L1 cells (Mouse embryonic fibroblast, ATCC) were cultured in DMEM supplemented with 10% bovine calf serum and 25 mM glucose. To induce the differentiation of 3T3-L1 preadipocytes, two days of post-confluence cells were treated with complete differentiated media (CDM) which contains 100 nM insulin, 400 nM cortisone or 1 μM dexamethasone, 500 μM IBMX (isobutylmethylxanthine) with or without 1 μM of CNX-010-49. Media was changed for every 2 days with fresh media containing 100 nM insulin and CNX-010-49 till day 6. On day 7 cells were cultured in regular culture media and after 24 h, triglyceride content of the cells were estimated (Diagnostic systems, Germany). One set of cells were processed for Oil red O staining. Treated cells were washed twice with phosphate-buffered saline (PBS), fixed in 10% formalin for 1 h and washed once with 60% isopropanol. Cells were then stained with 60% of filtered oil red O stock solution (0.35 g of oil red O (Sigma, USA) in 100 ml of 100% isopropanol) for 15 min. Cells were washed thrice with water for 5 min each and later visualized under microscope.

For the measurement of adipocytes hypertrophy, mature adipocytes were treated with media containing GPCI (25 mM glucose, 500 μM palmitate, 500 nM cortisone, 10 ng/ml of TNFα, IL-1β & IFγ) with or without 1 μM of CNX-010-49 for 48 h. Post 48 h, cells were lysed and triglyceride content of the cells was estimated (Diagnostics system, Germany).

For lipolysis assay, fully differentiated 3T3-L1 cells (mature adipocytes) were treated with 1 μM of isoproterenol and 100 nM cortisone with or without 1 μM CNX-010-49 for 18 h. Glycerol released in the medium was assayed using Free Glycerol Reagent (Sigma, USA). Lipolysis data was normalized to total protein.

### Isolation, culturing and hepatic glucose release in primary hepatocytes

Hepatocytes from Swiss albino mice were prepared as per the method of Seglen [33]. Hepatocytes were collected by centrifugation at 300 rpm for 3 min at 4°C. The viability of the hepatocytes was measured by Trypan blue exclusion and then seeded onto collagen coated 6 well/96-well tissue culture plates in DMEM containing 20% FBS and 10 mM Nicotinamide and maintained at 37°C in a humidified atmosphere of 5% CO_2_. After 3 h of attachment, the medium was replaced with fresh growth medium.

To measure glucose release from the hepatocytes, cells were treated with gluconeogenesis inducing media (glucose free DMEM media with 5 mM lactate, 5 mM pyruvate, 1 μM cortisone, 10 μM forskolin, 1% FBS with or without 1 μM of CNX-010-49) for 18 h. Glucose released in the media was measured using GOD method (Diagnostic systems, Germany). Values were normalized to total protein. Also one set of treatment was processed for gene expression analysis.

### 11β-HSD1 cell-based enzymatic activity assay

CHO-K1 (ATCC) cells stably expressing human 11β-HSD1 gene (OriGene Technologies, USA) and fully differentiated subcutaneous human adipocytes (Zen-Bio, Inc, USA) were used for determination of half-maximum inhibitory concentration (IC_50_) of CNX-010-49 towards human 11β-HSD1 isoform. Fully differentiated C2C12 cells (expressing native 11β-HSD1 protein) were used to determine IC_50_ towards the mouse isoform.

For determination of IC_50_, CHO-K1 cells were seeded in a serum free Ham’s F-12 media containing 400 nM cortisone and 500 μM NADPH with or without CNX-010-49. Inhibitor was dissolved in DMSO and diluted in media serially to get different concentration to determine IC_50_ (8 concentrations were used starting from 0.001 nM to 3 μM). Post 18 h treatment, the culture media was analyzed for the inhibition of cortisone to cortisol conversion using LCMS/MS (ABI-4000 QTRAP). Similar protocol was followed with C2C12 cells to determine IC_50_ (from 1 nM to 3 μM) for mouse isoform.

### HSD–related enzymes cell-based activity assay

CHO-K1 cells transiently expressing human 11β-HSD2 and 17β-HSD3 gene (OriGene Technologies, USA) and T47D (ATCC) breast cancer cell line that expresses 17β-HSD1 were used to determine inhibitory constants towards the respective enzymes.

For determination of inhibition towards HSD–related enzymes; cortisol was used as substrate for 11β-HSD2 (which will be converted to cortisone), androstenedione for 17β-HSD3 (converted to testosterone) and estrone for 17β-HSD1 (converted to estradiol). Post 18 h of treatment, the culture media was analyzed and inhibition of respective substrate conversion was analyzed using LCMS/MS (ABI-4000 QTRAP).

### 11β-HSD1 reductase and dehydrogenase enzymatic assay

N-terminal deleted human 11β-HSD1 pure protein (cloned, expressed using pET27b vector in bacteria and later purified in-house using Ni-NTA agarose beads) was used to evaluate both reductase and dehydrogenase enzymatic activity. For reductase activity, the reaction buffer contained 200 nM cortisone and 500 μM NADPH. For dehydrogenase activity, the reaction buffer contain 200 nM cortisol and 500 μM NADP. After 4 h incubation at 37°C, converted cortisol or cortisone was analyzed using LCMS/MS (ABI-4000 QTRAP).

### *In vivo* efficacy studies in C57BL/6j mice on high fat diet

Six week old male C57BL/6 J mice were housed 2 per polypropylene cage, maintained at 23 ± 1°C, 60 ± 10% humidity, exposed to 12 h cycles of light and dark. Control group (n =8) animals were fed a standard chow diet (D10001; 10% kcal from fat); HFD (high fat diet) group (n = 16) fed high fat diet (D12492; 60% kcal from Research Diets, Inc., New Jersey, USA). After 11 weeks of diet intervention, the HFD fed animals were randomized to either vehicle (HFD control) or CNX-01-49 (30 mg/kg, orally twice a day in 0.5% Carboxy methyl cellulose) treatment groups (n = 8) based upon body weight, glucose AUC during OGTT and fasting blood glucose. The treatment was further continued for another 10 weeks. Body weight, glucose in fasting (6 h) and fed state, triglycerides in fasting (12 h) state were measured weekly and plasma insulin was measured at the end of study. At the end of the study, blood was collected for determination of plasma glycerol and leptin levels following which the mice were euthanized under isoflurane anaesthesia and necropsied. Adipose tissues were weighed and a portion was immediately collected in formalin and processed for histological examination. Animal experiment protocols and experimental procedures were approved by the Connexios Institutional Animal Ethics Committee which is in accordance with the ARRIVE guidelines (Additional file 1) [34].

### Quantitative PCR analysis

At the end of treatment periods, total RNA was extracted from different tissues using TRIZOL. Equal amount of RNA was reverse transcribed and later amplified using specific primers. Beta-actin was used as an internal control for the quantitative analysis. The primer sequence is available upon request.

### DNA isolation and quantitative real-time PCR assay for mitochondrial copy number

DNA from C2C12 myotubes was isolated using Phenol extraction method. Quantitative real-time PCR was performed for mtND1 (mitochondrial gene) using DNA as template and normalized to HPRT (nuclear gene) gene.

### Blood and plasma assay

Blood glucose was measured using hand-held Accu-check glucometer (Roche Diagnostics, Germany). An ultra-sensitive insulin enzyme-linked immunosorbent assay (ELISA) kit (Downers Grove, USA) was used to determine plasma insulin levels. Circulating serum TG, plasma glycerol and leptin were estimated using the TG estimation kit (Diagnostic systems, Germany), glycerol estimation kit (Sigma, USA) and Leptin estimation kit (R & D systems, USA) respectively as per the manufacturer’s instruction.

### *Ex vivo* 11β-HSD1 inhibition assay

The duration and percent inhibition of 11β-HSD1 activity by CNX-010-49 (30 mg/kg, single dose orally) in different tissues (liver, adipose, skeletal muscle and brain) in Swiss albino mice was evaluated by an *ex vivo* 11β-HSD1 inhibition assay. We have used 4 animals for every time point in the study. After dosing the animals orally, animals were sacrificed at each time point and different tissues (liver, skeletal muscle, adipose and brain) were removed, minced and incubated with cortisone for 16 h. Converted cortisol was measured by LCMS/MS (ABI-4000 QTRAP) and the percent inhibition of 11β-HSD1 activity relative to vehicle-treated controls was calculated.

### Oral Glucose Tolerance Test (OGTT)

After a 6 h fast, CNX-010-49 or vehicle was administered 60 min prior to administration of glucose (2 g/kg body wt) by oral gavage. Blood samples were collected from the tail vein 60 min before treatment and at 0, 10, 20, 30, 60, 90 and 120 min after glucose load for estimating glucose.

### Insulin tolerance test (ITT)

ITT was performed in 6 h fasted mice during 9th week of treatment. Blood samples were collected prior to and at 0, 15, 30 and 60 min after insulin (human R Insulin, Eli Lilly) administration (2 IU/kg, i.p.) for glucose estimation.

### Pyruvate tolerance test (PTT)

Pyruvate Tolerance Test (PTT) was performed in overnight fasted mice during 10th week of treatment. Blood samples were collected from the tail vein 60 min before treatment and at 0, 15, 30, 60, 90 and 120 min after pyruvate load (2 g/kg, intraperitonial) for estimating glucose. The data was analyzed for change in glucose levels in CNX-010-49 treated animals when compared with HFD control.

### Measurement of thermogenesis

For the cold exposure experiment, mice were housed individually and transferred to a cold environment with an ambient temperature of 4°C. Body temperature was assessed at the end of treatment period using a rectal probe. Temperature was measured for every 15 min for 60–75 min and animals were then brought to room temperature.

### Estimation of liver TG

Liver TG was extracted according to Folch’s method. The TG from liver homogenate was extracted with chloroform: methanol (2:1) mixture, the organic layer dried in a speed vac, re-suspended in isopropyl alcohol and liver TG content was analyzed by using TG estimation kit (Diagnostic systems, Germany).

### Adipose tissue collection, immunofluorescence and image analysis

Subcutaneous adipose tissue was removed and was fixed in 10% buffered neutral formalin. It was then embedded in paraffin, sectioned and stained. All adipose sections were viewed at 40X magnification, and images were captured using Zeiss microscope connected via camera to a computer (progres® capture pro 2.1 camera). Adipocytes size measurement was performed using a computer-assisted image analysis progres® capture pro software. From each animal 10 images were measured. The area of each adipocyte was measured by tracing the cell boundary on the images captured. All adipocytes that had complete cell boundary were measured. Data obtained after measurement were averaged to individual animal and later group mean and SEM were calculated.

For PRDM16 and UCP1 immuno staining, subcutaneous adipose sections were de-paraffinized and blocked in 1% BSA in PBS for 30 min at RT and incubated with PRDM16 (ProSci, USA) or UCP1(Abcam, USA) primary antibodies for 90 min at RT, followed by washing with PBS. Sections were then incubated with secondary antibody (goat anti-rabbit Alexa Fluor 555 secondary antibody, Molecular Probes, Invitrogen) for 30 min at RT and mounted using fluromount. Localization of PRDM16 and UCP1 were assessed and the images were captured at 40X magnification.

### Assessment of JNK and eIF2α phosphorylation

At the end of treatment period, 10 mg of muscle (soleus) and subcutaneous adipose tissues were collected from each animal in respective groups. Lysates were prepared by homogenization and 50 μg lysate from each group was subjected to SDS-PAGE, transferred onto nitrocellulose membranes and probed with antibodies against phospho-JNK, phospho-eIF2α, total JNK and Actin (Cell signaling, USA) and developed by enhanced chemiluminescence (West Pico, Thermo Scientific, USA).

### Estimation of serum biomarkers

Serum was collected at the end of the study and ELISA (Enzyme Linked Immunosorbent Assay) was performed as per the kit protocol to measure changes in leptin (R&D system), PAI-1 (Invitrogen), Fetuin-A (Uscn Life Science Inc) and IL-6 (R&D systems) levels in the serum.

### Statistical analysis

Prism 5.01 software (GraphPad Software, San Diego CA) was used for statistical analysis. All the values are expressed as Mean ± SEM and ‘n’ indicates the number of animals in each group. When only two groups were analyzed, statistical comparison was conducted by One-way ANOVA followed by Dunnett’s test. Repeated measure based parameters (such as weekly fasting glucose, serum triglycerides and body weight) were analyzed using two-way ANOVA for repeated measures followed by Bonferroni correction (with time and diet/inhibitor treatment as factors). Statistical details (p-value, F, and degree of freedom (Df)) are provided in the figure legends along with the results of the two-way ANOVA testing. P < 0.05 was considered statistically significant.

## Results

### CNX-010-49 is a highly potent, selective inhibitor and has a good pharmacodynamic activity

**Figure 1:**
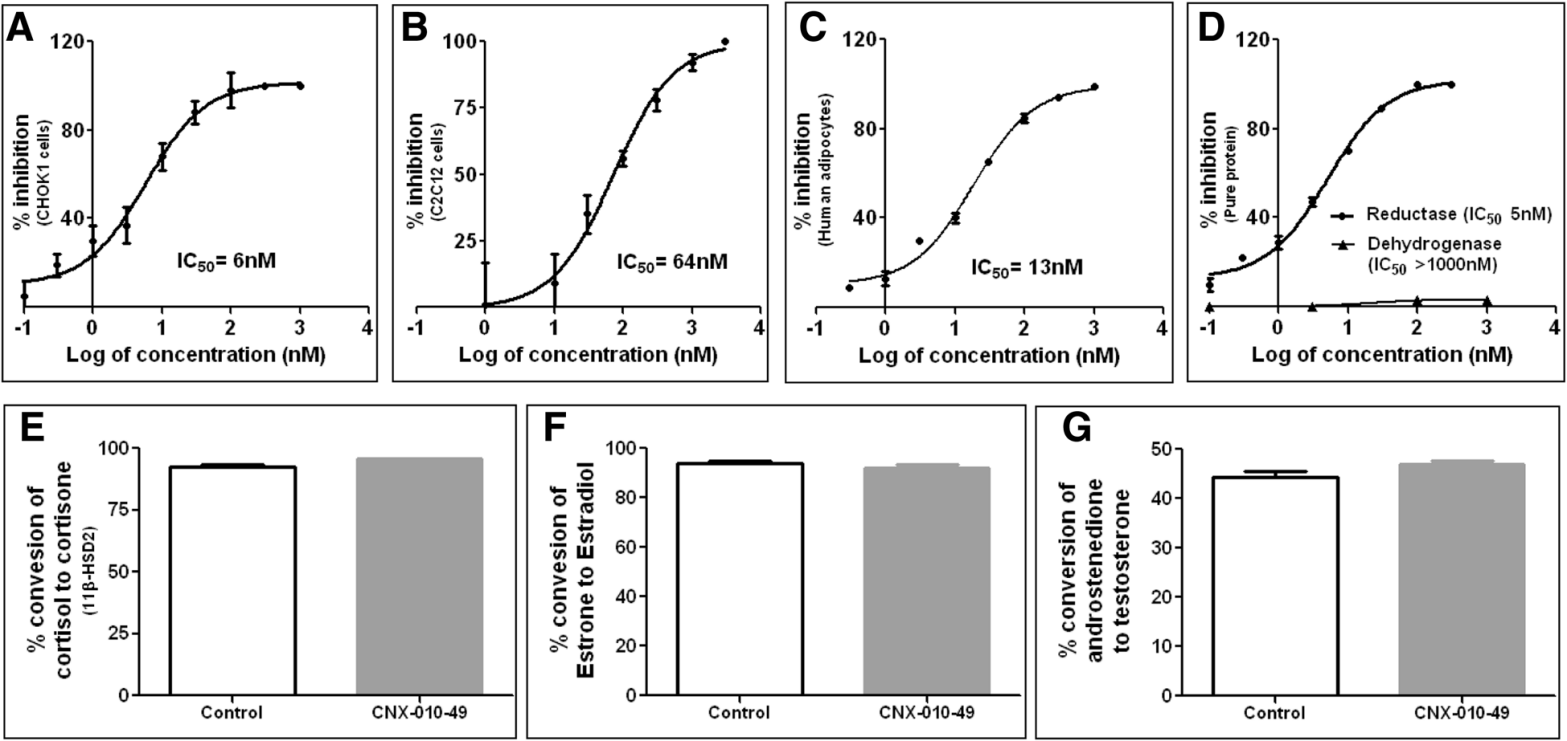
**Potency and selectivity of CNX-010-49.** Inhibition curve with increasing concentrations of CNX-010-49 for reductase activity in CHOK1 cells stably expressing human 11β-HSD1 **(A)**, Mouse C2C12 cells **(B)**, fully differentiated human adipocytes **(C)**, both reductase and dehydrogenase activity with recombinant human 11β-HSD1 protein **(D)** as mentioned in the materials and methods. Selectivity of CNX-010-49 (100 μM) against HSD related enzymes were evaluated in CHOK1 cells transiently over expressing human 11βHSD2 and 17β HSD3 respectively **(E & G)** and T47D cells for 17βHSD1 **(F)**. Data are means of three individual experiments with standard deviations (n = 6).

CNX-010-49 is a highly potent 11β-HSD1 inhibitor towards both human and mouse isoforms with an IC_50_ of 6 nM and 64 nM respectively (Figure 1A & B) as established from cells over expressing human 11β-HSD1 in CHO-K1 cell and fully differentiated mouse C2C12 myotubes respectively. IC_50_ for CNX-010-49 in primary human mature adipocytes is 13 nM (Figure 1C). CNX-010-49 inhibits reductase activity more than dehydrogenase activity (>100X, Figure 1D). CNX-010-49 is highly selective inhibitor with no inhibition up to 100 μM towards 11β-HSD2, 17β-HSD1 and 17β-HSD3 (Figure 1E, F & G). In a single dose pharmacodynamic activity evaluation in Swiss albino mice, oral administration of CNX-010-49 at 30 mg/kg inhibited 11β-HSD1 activity by 58% and 24% at 1 h and 7 h respectively in liver. In adipose, the inhibition was 41% at 1 h and continued to be same till 7 h. In skeletal muscle, the inhibition was ~38% at 1 h and 7 h. At end of 24 h, there was no inhibition of 11β-HSD1 observed (data not shown). However there was no inhibition observed in brain (Table 1).

**Table 1:** Effect of CNX-010-49 on pharmacodynamic activity

	**Liver**		**Skeletal muscle**		**Adipose**		**Brain**	
**Time (h)**	**1**	**7**	**1**	**7**	**1**	**7**	**1**	**7**
% inhibition with CNX-010-49	58 ± 2	24 ± 6	41 ± 4	44 ± 5	38 ± 7	42 ± 5	0	0

After oral administration of CNX-010-49 at 30 mg/kg (single dose) to Swiss albino mice (n = 4 per time point), pharmacodynamic activity of CNX-010-49 at different time points was measured in liver, muscle, adipose and brain tissues *ex vivo* as described in Methods.

### CNX-010-49 decreases liver gluconeogenic activity in primary cultures of mouse hepatocytes and hence has a potential to control fasting glucose

**Figure 2:**
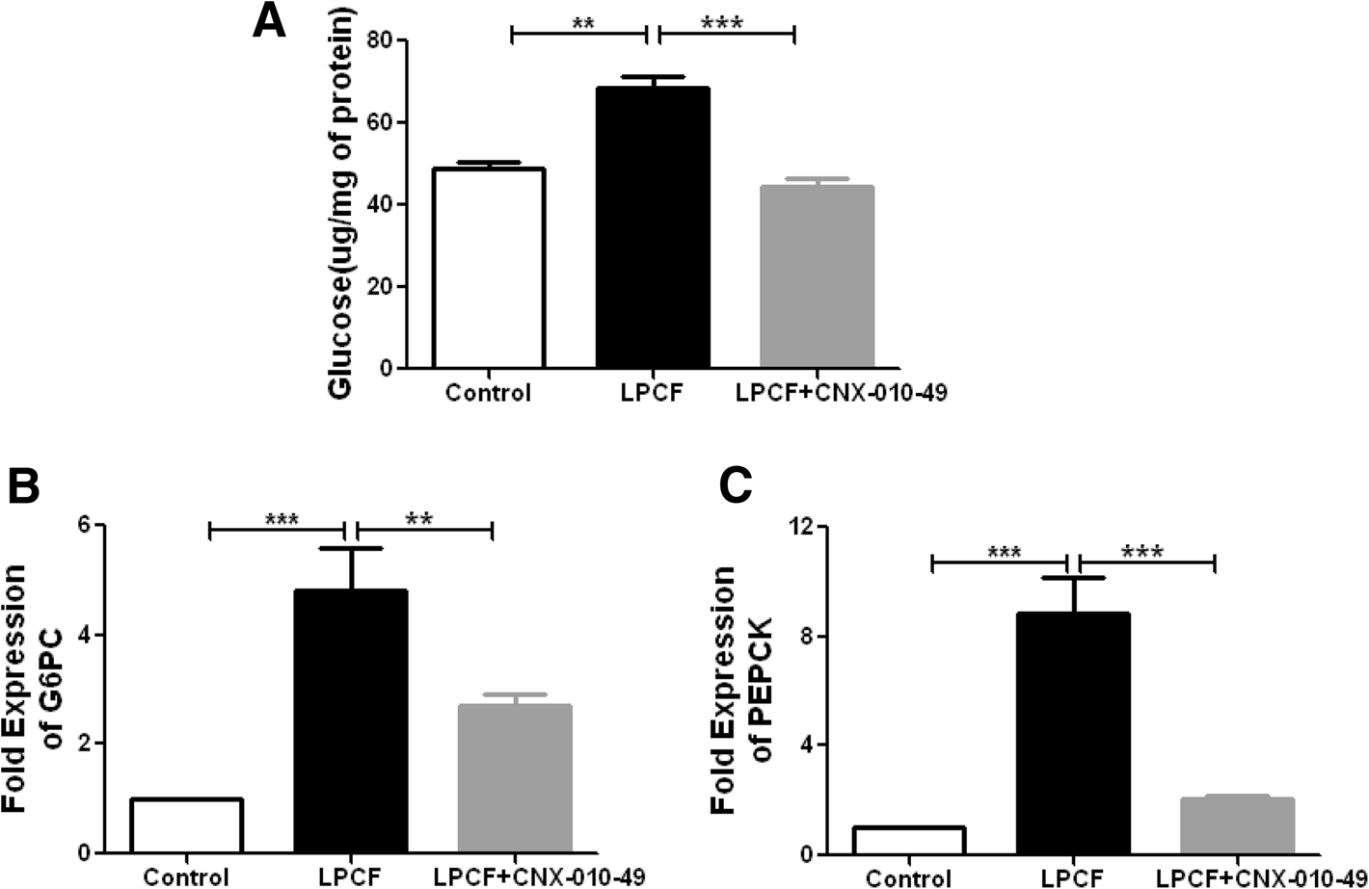
**Effect of CNX-010-49 on liver gluconeogenesis in primary mouse hepatocytes.** Mouse primary hepatocytes were treated with glucose free media containing LPCF (5 mM Lactate, 5 mM Pyruvate, 1 μM cortisone, 10 μM of forskolin) with or without 1 μM of CNX-010-49 for 18 h. Post 18 h, glucose released in the media **(A)** and mRNA expression gluconeogenic markers G6PC and PEPCK **(B & C)** were measured as mentioned in Methods. All the values are expressed as Mean ± SEM. Statistical comparison was conducted by One-way ANOVA followed by Dunnett’s test (n = 6) (*P < 0.05, **P < 0.01 and ***P < 0.001).

In the presence of gluconeogenic substrates (lactate and pyruvate) along with signaling molecules (forskolin and glucocorticoids), mouse hepatocytes showed a significant increase in hepatic glucose release along with increase in mRNA expression of both G6PC (glucose-6-phosphatase, catalytic subunit) and PEPCK (phosphoenolpyruvate carboxykinase) (5 and 9 fold increase respectively against control cells) which are the key mediators of hepatic glucose production. Inhibition of 11β-HSD1 by 1 μM of CNX-010-49 significantly reduced hepatic glucose release by ~45% and mRNA expression of both G6PC and PEPCK (40% and 70% respectively) indicating a potential of CNX-010-49 to reduce fasting glucose (Figure 2A-C).

### CNX-010-49 increases muscle glucose oxidation, reduces muscle proteolysis and increases mitochondrial biogenesis

**Figure 3:**
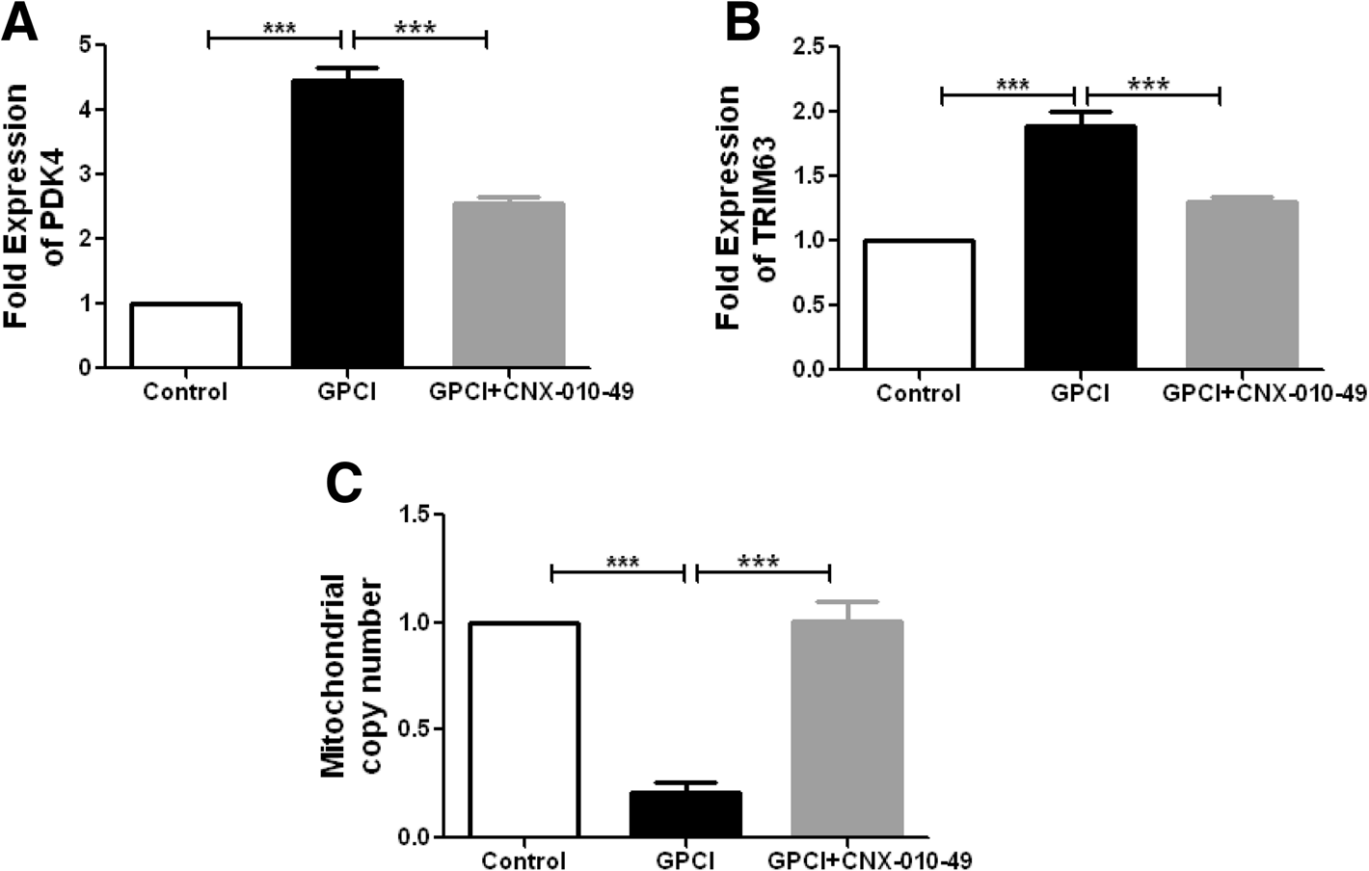
**Effect of CNX-010-49 on muscle glucose oxidation, proteolysis and mitochondrial biogenesis.** C2C12 myotubes were treated with GPCI (25 mM Glucose, 500 μM Palmitate, 1 μM of Cortisone, 10 ng/ml of each inflammatory cytokines (TNFa, IL1 β and IFNγ)) for 18 h with or without 1 μM of CNX-010-49. Post 18 h, mRNA expression of PDK4 and TRIM63 **(A & B)** and mitochondrial gene ND1 **(C)** were measured as mentioned in Methods. All the values are expressed as Mean ± SEM. Statistical comparison was conducted by One-way ANOVA followed by Dunnett’s test (n = 6) (*P < 0.05, **P < 0.01 and ***P < 0.001).

In C2C12 myotubes, the presence of excess free fatty acids, inflammatory cytokines and cortisone (*in vitro* disease mimicking condition); PDK4 (pyruvate dehydrogenase kinase 4) mRNA expression was increased by more than 4 fold as compared to vehicle control. Inhibition of 11β-HSD1 by CNX-010-49 reduced PDK4 expression by 40% (Figure 3A). Under the similar condition, we observed around 2 fold increase in mRNA expression of TRIM63 (tripartite motif containing 63; an E3 ubiquitin-protein ligase which is known to enhance muscle proteolysis) as compared to vehicle control. Treatment with CNX-010-49 reduced the expression by 30% (Figure 3B). Mitochondrial copy number was decreased by ~75% under disease mimicking condition compared to vehicle control. Inhibition of 11β-HSD1 by CNX-010-49 restored the mitochondrial number (Figure 3C).

### CNX-010-49 inhibits both adipocytes differentiation, lipolysis and hypertrophy

**Figure 4:**
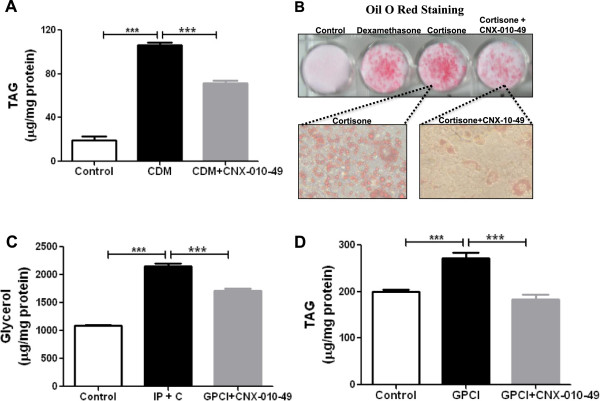
**Effect of CNX-010-49 on adipocytes differentiation, hypertrophy and lipolysis.** Mouse 3T3-L1 preadipocytes were differentiated into mature adipocytes using complete differentiating media (CDM) in the presence and absence of 1 μM of CNX-010-49. The extent of inhibition of adipocytes differentiation was assessed by measuring triglyceride levels **(A)** and Oil red O staining **(B)**. For inhibition of lipolysis, mature adipocytes were treated with 1 μM isoproterenol and 100 nM cortisone with or without 1 μM of CNX-010-49 for 18 h. Post 18 h, glycerol released into the media was measured **(C)**. For inhibition of adipose hypertrophy, mature adipocytes were treated with GPCI (25 mM glucose, 500 μM palmitate, 1 μM cortisone, 10 ng/ml of inflammatory cytokines (TNFa, IL1 β, IFNγ)) for 48 h with or without 1 μM of CNX-010-49. Post 48 h, triglyceride accumulation was measured **(D)**. All the values are expressed as Mean ± SEM. Statistical comparison was conducted by One-way ANOVA followed by Dunnett’s test (n = 6) (*P < 0.05, **P < 0.01 and ***P < 0.001).

Conversion of inactive cortisone to active cortisol by 11β-HSD1 favors adipogenesis. CNX-010-49 treatment reduced the differentiation/adipogenesis capacity by 34% as measured by accumulation of triglycerides in adipocytes as well as by oil red O staining (Figure 4A & B). Both cortisone and isoproterenol stimulated lipolysis by 2 fold in mature 3T3-L1 adipocytes as measured by the release of glycerol in the culture medium. CNX-010-49 treatment reduced adipocytes lipolysis by 25% (Figure 4C). Excess metabolic overload and inflammation mediated hypertrophy of adipocytes as measured by enhanced triglyceride accumulation, was reduced by 35% with CNX-010-49 treatment (Figure 4D).

### CNX-010-49 reduces fasting blood glucose in DIO mice

**Figure 5:**
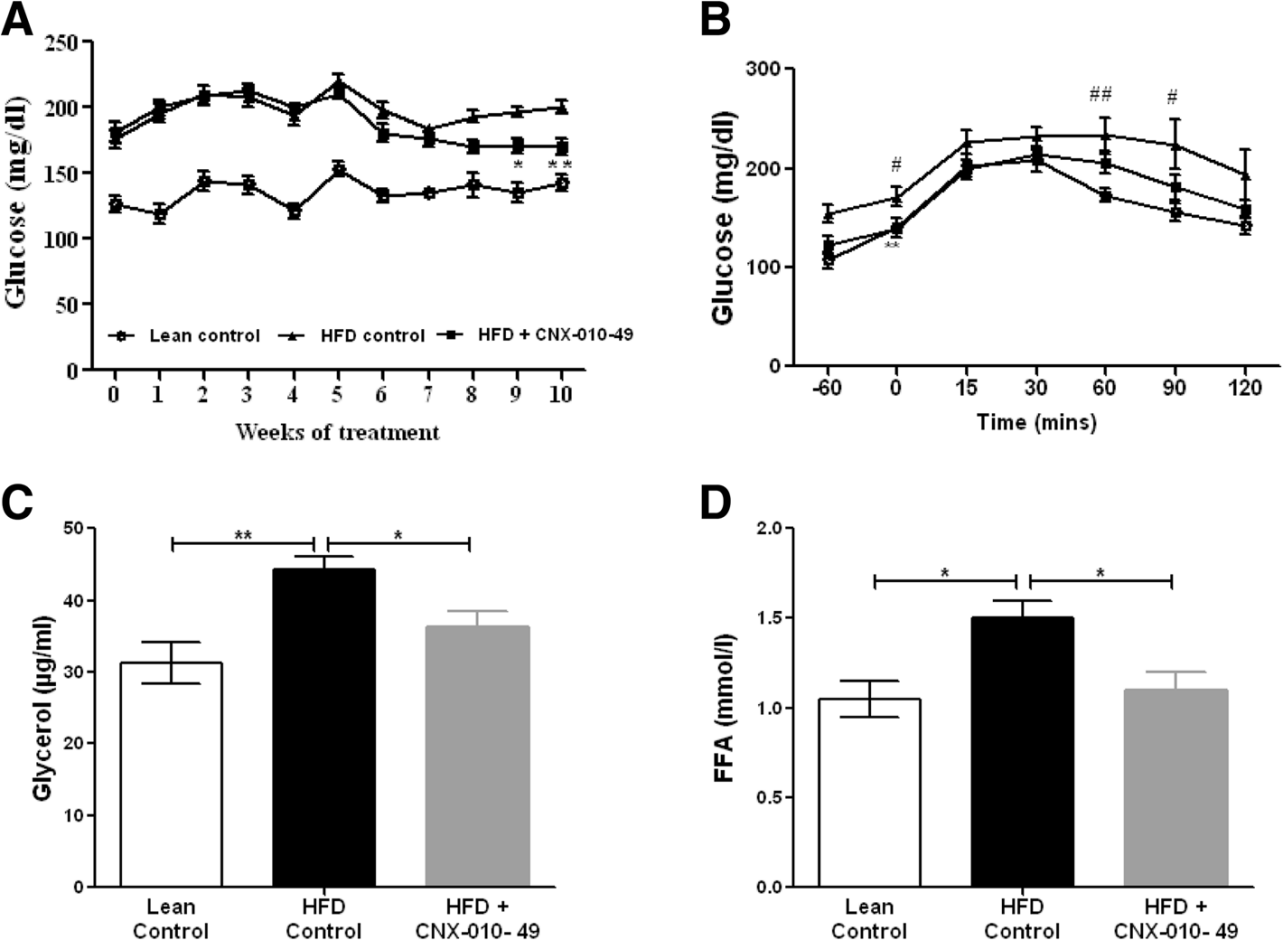
**Effect of CNX-010-49 on fasting glucose in DIO mice of HFD.** Fasting Glucose levels **(A)** were monitored weekly. To measure the gluconeogenic state of liver, pyruvate tolerance test **(B)** was performed as described in the Methods. At the end of the treatment period, serum glycerol **(C)** and free fatty acids **(D)** were measured as described in the Methods. Data in all panels are mean ± SEM (n = 8 mice/group). Statistical comparison was conducted by One-way ANOVA followed by Dunnett’s test or repeated measures ANOVA followed by Bonferroni correction **(A & B)** (n = 8 mice/group). Two-way repeated measures ANOVA indicated that CNX-010-49 significantly reduced the fasting glucose (p = 0.01; F = 5.08; Df = 22) (# - significance of HFD control against lean control, *- significance of CNX-010-49 treatment against HFD control) (*P < 0.05, **P < 0.01 and ***P < 0.001).

Compared to the lean control animals the HFD control animals exhibited a significant increase in fasting glucose (136 ± 6 vs. 200 ± 8 mg/dl; P < 0.001) during the entire study period, indicating development of insulin resistance and hyperglycemia (Figure 5A). In CNX-010-49 treated animals, fasting glucose started decreasing from week 5 of treatment and by the end of treatment period reached a significant 15% reduction (170 ± 6 vs 200 ± 5 mg/dl; P < 0.01) when compared with the HFD control mice.We evaluated the gluconeogenic state of liver before terminations of the study by performing a pyruvate tolerance test where animals were challenged with gluconeogenic substrate pyruvate and glucose levels in serum was measured. Compared to lean control animals, HFD animals showed an increase in serum glucose AUC (20736 ± 1013 vs 26511 ± 2057) upon pyruvate challenge. When compared with HFD control animals, CNX-01-49 treatment resulted in significant decrease in glucose AUC (26511 ± 2057 vs 22775 ± 97) amounting to a ~15% suggesting decreased gluconeogenesis (Figure 5B).The HFD control animals showed an ~25% and ~50% increase in fasting glycerol and free fatty acid levels respectively when compared to lean control animals. CNX-010-49 treated animals showed ~18% and 20% (P < 0.05) decrease in fasting glycerol and free fatty acids respectively (Figure 5C & D). This indicates that treatment with CNX-010-49 modulates adipocytes physiology and reduces supply of pro-gluconeogenic substrates to the liver.

### CNX-010-49 improves glucose tolerance in DIO mice

**Figure 6:**
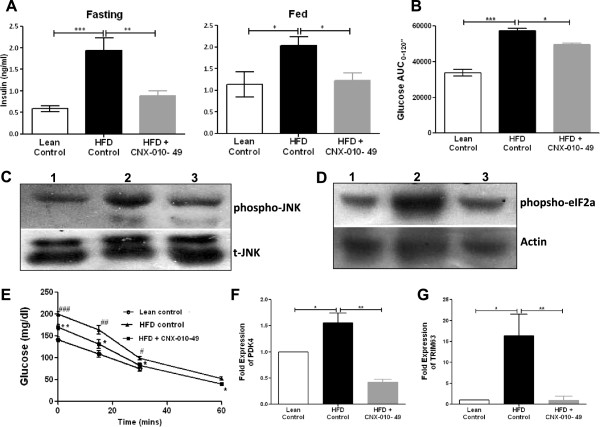
**Effect of CNX-010-49 on insulin sensitivity, hyperinsulinemia and glucose clearance in DIO mice on HFD.** At the end of the treatment period, fasting and fed insulin levels **(A)** and OGTT was performed **(B)** by oral administration of glucose (2 g/kg); plasma glucose was measured and AUC was calculated. Data in all panels are mean ± SEM (n = 8/group). At the end of the treatment, adipose and muscle tissue samples were collected from Lean control (1), HFD Control (2) and CNX-010-49 treated animals (3) and subjected to Western blots for p-JNK/total JNK **(C)** for muscle and p-EIF2α/Actin **(D)** for adipose. The blots are representative date from five animals from each group. ITT **(E)** was performed in mice to determine the effect of CNX-010-49 on glucose clearance as mentioned in the Methods. mRNA expression levels of muscle PDK4 and TRIM63 **(F & G)** were measured as mentioned in Methods. Statistical comparison between control was conducted by One-way ANOVA followed by Dunnett’s test (n = 8 mice/group) (# - significance of HFD control against lean control, *- significance of CNX-010-49 treatment against HFD control) (*P < 0.05, **P < 0.01 and ***P < 0.001).

To examine whether glucose intolerance in HFD mice was improved by the CNX-010-49 treatment, an oral glucose tolerance test was performed at the end of the treatment. Upon oral glucose challenge, a significant increase in the glucose excursion in HFD mice were observed when compared to lean control (AUC of 57376 ± 1382 in HFD control vs 33803 ± 1919 in lean control; P < 0.001). However CNX-010-49 treatment showed ~13% decrease in glucose levels (49680 ± 734 in treatment vs 57376 ± 1382 in HFD control; P < 0.05) indicating the potential of CNX-010-49 to reduce post prandial hyperglycemia (Figure 6B).

### CNX-010-49 reduces hyper insulinemia and improves peripheral insulin sensitivity

In comparison with the lean control animals, HFD control animals showed significant increase in fasting insulin levels (0.59 ± 0.07 vs 1.94 ± 0.29 ng/ml; P < 0.001). Treatment with CNX-010-49 significantly lowered plasma insulin levels by ~40% (0.89 ± 0.11 vs 1.94 ± 0.29 ng/ml; P < 0.05) when compared with the hyperinsulinemic HFD animals (Figure 6A). A similar trend was observed with fed insulin. Along with reduced insulin levels, insulin sensitivity was significantly improved in CNX-010-49 treated animals as evident from improved insulin tolerance test, reduced skeletal muscle phospho-JNK and reduced subcutaneous adipose phospho-eIF2α levels (Figure 6C, D & E). Also we observed a significant decrease in mRNA expressions of PDK4 and TRIM63 in muscle tissue of CNX-010-49 treated animals indicating a possible improvement in glucose oxidation (Figure 6F & G).

### CNX-010-49 improves lipid profile in DIO mice

**Figure 7:**
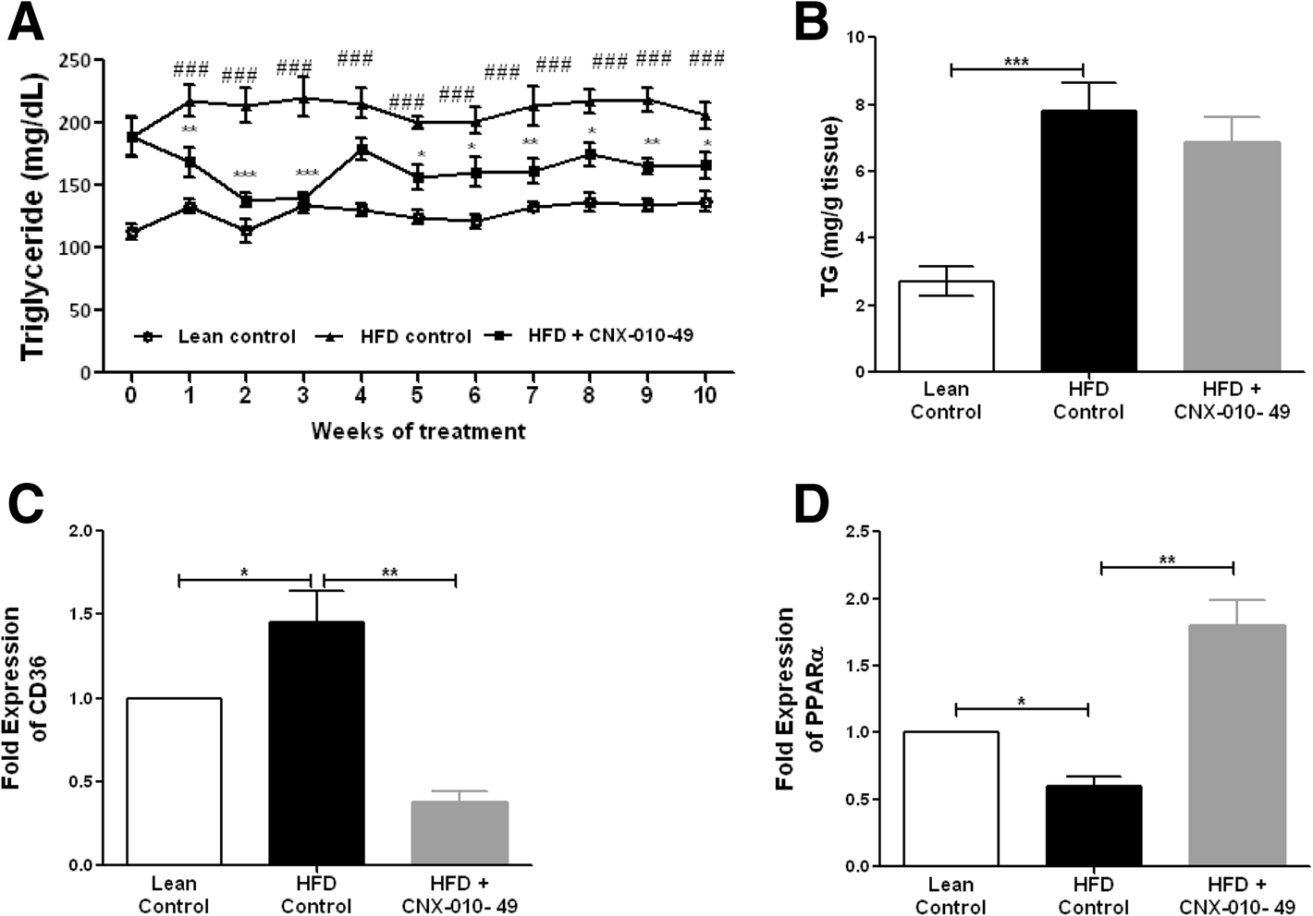
**Effect of CNX-010-49 on lipid profile in DIO mice.** Fasting serum TG levels **(A)** were monitored weekly. After the study termination, liver TG **(B)** was analyzed. Data in all panels are mean ± SEM (n = 8 mice/group). mRNA expression levels of liver CD36 and PPARα **(C & D)** were measured as mentioned in Methods. Statistical comparison was conducted by One-way ANOVA followed by Dunnett’s test or repeated measures ANOVA followed by Bonferroni correction **(A)** (n = 8 mice/group). Two-way repeated measures ANOVA indicated that CNX-010-49 significantly reduced the Fasting serum TG (p = 0.001; F = 1.865; Df = 20). (# - significance of HFD control against lean control, *- significance of CNX-010-49 treatment against HFD control) (*P < 0.05, **P < 0.01 and ***P < 0.001).

The effect of treatment on circulating TG levels is represented in Figure 7A.In HFD control animals, the TG was increased by ~1.7 fold (210 ± 8 mg/dl; P < 0.001) when compared to lean control (128 ± 8 mg/dl) during the study period. On the other hand, the HFD fed animals treated with CNX-010-49 showed ~20% reduction in TG levels (165 ± 9 mg/dl; P < 0.001) when compared to HFD control. The decrease in plasma TG upon CNX-010-49 treatment was observed from week 1 and was maintained throughout the study period. Also CNX-010-49 treatment has shown a non-significant reduction in the liver TG levels as compared to HFD control (Figure 7B).We analyzed expression of fatty acid transporter CD36 and PPARα which is involved in fat oxidation in liver tissue. As expected expression of CD36 increased by ~1.5 fold and a significant decrease in PPARα in HFD animals as compared to lean control indicating increased fat uptake and decreased fatty acid oxidation. Treatment with CNX-010-49 showed a decrease in CD36 and increased PPARα expression indicating a decreased fat uptake and improved fat oxidation in liver as evident from decreased liver and serum TG levels (Figure 7C & D).

### CNX-010-49 has a potential to improve thermogenesis and reduce body weight

**Figure 8:**
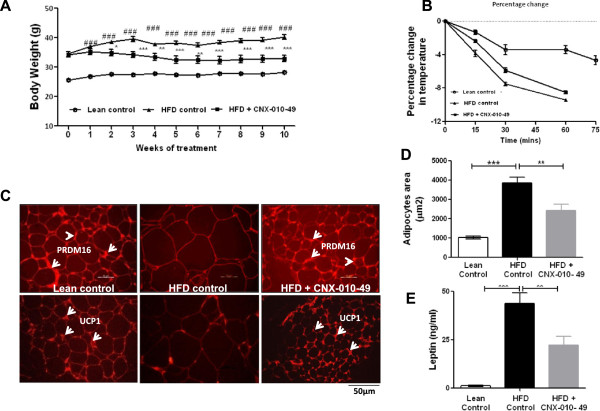
**Effect of CNX-010-49 on thermogenesis and body weight in DIO mice.** Body weight was measured weekly **(A)**. Non-shivering thermogenesis was measured as described in the methods **(B)**. Immunohistochemistry of PRDM16 and UCP1 **(C)** in subcutaneous adipose tissue sections was performed as mentioned in the Methods. Subcutaneous adipocytes size **(D)** and serum leptin **(E)** levels were measured at the end of treatment. Statistical comparison was conducted by One-way ANOVA followed by Dunnett’s test or repeated measures ANOVA followed by Bonferroni correction **(A)** (n = 8 mice/group). Two-way repeated measures ANOVA indicated that CNX-010-49 significantly reduced body weight (p = 0.001; F = 14.02; Df = 22). (# - significance of HFD control against lean control, *- significance of CNX-010-49 treatment against HFD control) (*P < 0.05, **P < 0.01 and ***P < 0.001).

In the lean control animals, a gradual increase in body weight was observed during the 10 weeks study period (25.6 g to 28.1 g). In contrast, body weight of HFD control animals showed a rapid increase and recorded ~18% increase by end of the study (34.7 g to 41 g). Treatment with CNX-010-49 prevented the weight gain by ~20% (Figure 8A) without any change in the feed consumption (data not shown). We did not observe any significant increase in body weight from week 1 rather CNX-010-49 treatment decreased the body weight gradually till week 5 and later maintained the same during complete treatment period.Histological analysis of white adipose tissue revealed an increase in adipocytes size in HFD animals compared to that of lean control. Treatment with CNX-010-49 reduced adipocytes size significantly by ~30% (Figure 8C & D). Compared to HFD control animals, CNX-010-49 treatment enhanced non-shivering thermogenesis (Figure 8B). We observed a significant decrease in markers of brown fat phenotype and thermogenesis; positive regulatory domain containing 16 (PRDM16) and uncoupling protein 1 (UCP1) in HFD control adipocytes. CNX-010-49 treatment restored both PRDM16 and UCP1 protein expression thus shown a potential to enhance brown fat phenotype and thermogenesis (Figure 8C).Serum leptin levels were elevated in HFD control animals compared to lean control (43 ng/ml vs 1.24 ng/ml). Treatment with CNX-010-49 reduced serum leptin levels significantly by ~50% (22 ng/ml vs 43 ng/ml) (Figure 8E) in comparison with the HFD control animals.

### CNX-010-49 has a potential reduce markers associated with cardio vascular risks

**Figure 9:**
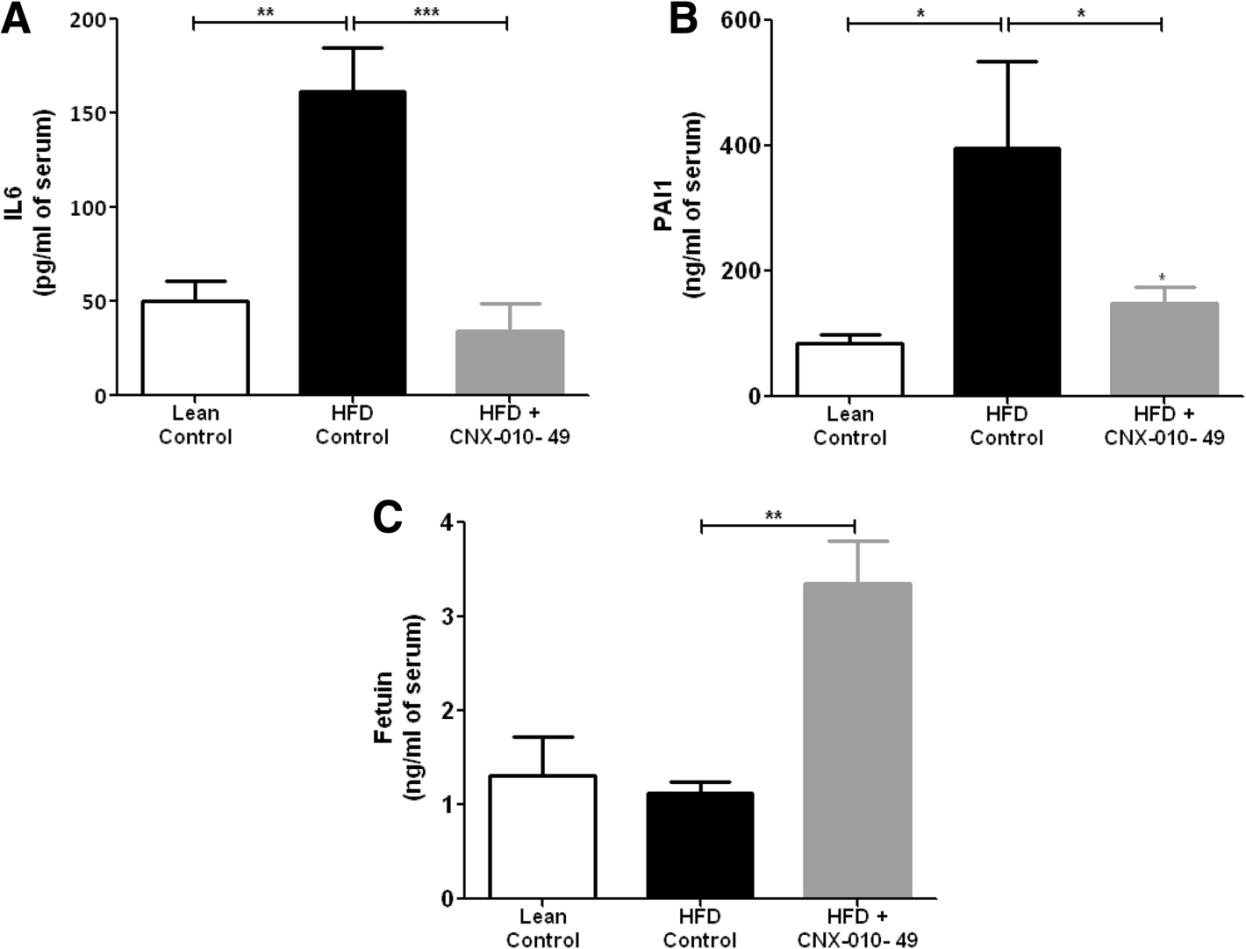
**Effect of CNX-010-49 on markers associated with cardio vascular risks.** At the end of the treatment, **(A)**, PAI1 **(B)** and FetuinA **(C)** levels in the serum were measured by ELISA. All the values are expressed as Mean ± SEM. Statistical comparison was conducted by One-way ANOVA followed by Dunnett’s test (n = 8 mice/group) (*P < 0.05, **P < 0.01 and ***P < 0.001).

Serum IL6 and PAI-1 levels (markers that are involved in stress and cardio protection) in HFD control animals were elevated significantly by 3 fold (161 pg/ml vs 50 pg/ml) and 3.5 fold (375 ng/ml vs 94 ng/ml) respectively compared to lean controls. Also we observed a non-significant decrease (~30%) in serum Fetuin-A levels in HFD control animals compared to lean controls. Treatment with CNX-010-49 decreased serum IL6 and PAI-1 levels significantly by ~75% (34 pg/ml vs 161 pg/ml) and ~60% (148 ng/ml vs 375 ng/ml) in comparison with HFD control animals. CNX-010-49 treatment increased serum Fetuin levels as compared to HFD control (3.2 ng/ml vs 1.2 ng/ml) (Figure 9A-C).

## Discussion

It is evident from multiple studies that glucocorticoids excess plays an important role in the development of metabolic syndrome, in particular T2DM [13]. Also it is confirmed from many recent studies that tissue specific glucocorticoids are involved in obesity and insulin resistance against the circulatory glucocorticoids [35-37].

The effect seen in these studies is an evidence to strengthen the understanding that tissue specific glucocorticoids play a major role in controlling metabolic syndrome. The pathology observed was not restricted to a particular tissue rather from multiple tissues. The therapeutic potential will be appreciated when we restrict/reduce the tissue level cortisol, an active glucocorticoid in multiple tissues.

In the present study, we have used a highly potent and selective 11β-HSD1 inhibitor CNX-010-49 to study the multiple aspects of the metabolic dysregulations as seen in the metabolic syndrome in DIO mice on HFD. CNX-010-49 is an orally dosable small molecule inhibitor of 11β-HSD1 which has a half-life of 7 h. It has shown a good tissue distribution and 11β-HSD1inhbition in multiple tissues like liver, skeletal muscle and adipose tissue. We did not observe brain 11β-HSD1 inhibition with CNX-010-49 indicating there is no safety concerns in terms of hypothalamic-pituitary-adrenal axis (HPA axis) activation [38]. Since several previous studies from both T2DM humans and rodents have demonstrated elevated tissue specific glucocorticoids [13,18-20], we did not measure the tissue cortisol levels in our study.

Gluconeogenesis is one of the basic features of hepatocytes. Abnormal gluconeogenesis will lead to both fasting and non-fasting glucose release in patients with type 2 diabetes. It is well established that 11β-HSD1 controls key gluconeogenic genes PEPCK and G6PC expression [39,40]. Our data clearly demonstrate that inhibition of hepatic 11β-HSD1 by CNX-010-49 suppress hepatic glucose output significantly along with reduced expression of PEPCK and G6PC in cultured mouse hepatocytes. DIO mice have shown a significant increase in fasting glucose which was inhibited by treatment with CNX-010-49. When challenged with pyruvate, CNX-010-49 treated animals had shown a reduction in glucose levels indicating reduced gluconeogenic state of liver. These data from both *in vitro* and *in vivo* clearly demonstrate the role of 11β-HSD1 in hyperglycemia and inhibition can give a potential benefit in controlling both fasting and non-fasting hyperglycemia.

Glucocorticoid excess is known to induce insulin resistance in skeletal muscle. This has a significant effect on the glucose clearance in the periphery. It has been shown that activity and expression of 11β-HSD1 in skeletal muscle has a significant impact on muscle insulin resistance [23]. Also recent studies have shown that insulin resistance can accelerate muscle protein degradation by activating ubiquitin-proteasome system [41]. Glucocorticoids are known to increase PDK4 [42,43] and TRIM63 (an E3 ubiquitin protein ligase also known as MuRF1) in skeletal muscle cells [44]. Treatment with CNX-010-49 reduced hyperinsulinemia (both fasting and fed plasma insulin levels) and improved peripheral insulin sensitivity as evident from improved insulin tolerance test which was further supported by reduced glucose intolerance. This improvement in insulin sensitivity was further supported by reduced phospho-JNK and phospho-eIF2α levels in skeletal muscle and adipose tissues of treated animals respectively. Treatment with CNX-010-49 reduced both PDK4 and TRIM63 expression along with restoration of mitochondrial copy number both *in vitro* and *in vivo*. In adipose tissue, treatment with CNX-010-49 inhibited lipolysis. Collectively these data suggests an overall improvement in the insulin sensitivity in the periphery.

Elevated tissue specific glucocorticoids are known to cause dyslipidemia and insulin resistance. They increase circulating triglyceride levels, hepatic *de novo* TAG synthesis and storage [45,46]. Elevated CD36 expression is known to increase hepatic triglyceride storage and secretion [47]. Serum TG levels were very high in the HFD control mice. Treatment with CNX-010-49 reduced serum TG significantly indicating the role of 11β-HSD1 in dyslipidemia. A non-significant reduction in hepatic triglyceride content was observed with CNX-010-49 treatment along with decreased CD36 expression indicating that reduced serum TG is not because of accumulation in liver. We also observed a significant increase in PPARα expression in liver indicating treatment might have increased fatty acid oxidation. It is reported in the literature that activation of PPARα reduces 11β-HSD1 expression and activity in liver [48].

The role of 11β-HSD1 in obesity is well established both by pharmacological interventions and also by knockout studies [28,30,32,49,50]. In agreement with the previous reports, we observed a significant decrease in body weight with CNX-010-49 treatment without altering the feed consumption rate (data not shown). CNX-010-49 treatment has reduced both adipocyte size and serum leptin levels significantly. Glucocorticoids are known to decrease non-shivering thermogenesis and favors lipid storage in adipose tissue along with significant decrease in UCP1 expression [51]. Recently it has been shown that PRDM16 induces brown fat phenotype in white adipose tissue [52]. Also it induces many other genes including UCP1 which are directly involved in uncoupled cellular respiration and energy expenditure [53]. Treatment with CNX-010-49 restores the expression of both PRDM16 and UCP1 in white adipose tissue. The relation between glucocorticoids and PRDM16 is still needs to be established.

Vascular calcification is known to be associated with cardiovascular disease. Recently it has been shown that serum Fetuin-A inhibits calcification [54,55]. Dexamethasone has been shown to suppress the expression of genes that inhibit calcification [56]. Elevated plasma levels of PAI-1 are known to associate with atherothrombosis and insulin resistance [57]. Glucocorticoids are known to increase the production of PAI-1 from adipose tissue [58]. Systemic chronic inflammation is a risk factor for cardiovascular disease where IL-6 act as central regulator [59]. CNX-010-49 treatment increased the serum Fetuin-A levels and decreased PAI-1 and IL-6 levels significantly indicating a potential cardiovascular benefit.

## Conclusions

In summary, CNX-010-49 is a selective and ‘pan’ tissue acting, orally bioavailable 11β-HSD1 inhibitor. Pharmacological inhibition of 11β-HSD1 with CNX-010-49 has normalized most of metabolic dysregulations like hyperglycemia, insulin resistance and dyslipidemia along with body weight.

CNX-010-49 is one of our lead compound in our discovery program. Further characterization is necessary before progressing to human studies.

## Abbreviations

11β-HSD1: 11β-hydroxysteroid dehydrogenase type1; DIO: Diet induced obesity; OGTT: Oral Glucose Tolerance Test; PAI-1: Plasminogen activator inhibitor-1; GCs: Glucocorticoids; UCP: Uncoupling protein; T2DM: Type 2 Diabetes Mellitus; TNFα: Tumor necrosis factor alpha; ALT: Aminotransferase; LDL: Low-density lipoprotein; IRS1: Insulin receptor substrate 1; IBMX: Isobutylmethylxanthine; HFD: High fat diet; ITT: Insulin tolerance test; PTT: Pyruvate tolerance test; G6PC: Glucose-6-phosphatase; PEPCK: Phosphoenolpyruvate carboxykinase 1; PDK4: Pyruvate dehydrogenase kinase 4; TRIM63: Tripartite motif containing 63; TG: Triglyceride; PRDM16: Positive regulatory domain containing 16; HPA axis: Hypothalamic-pituitary-adrenal axis.

## Competing interests

All the authors are employees of Connexios Life Sciences Pvt Ltd and declare that they have no competing interests.

## Authors’ contributions

KH, JS, NS, VSK, MNL, VS, CH, GVB, BSN, ASG, SP carried out experiments; TMA, AD, MKS, AMO, YM, MVV, SBP and JMR planned/executed the study and analyzed data. SBP wrote the manuscript. All authors read and approved the final manuscript.

## Pre-publication history

The pre-publication history for this paper can be accessed here:

http://www.biomedcentral.com/2050-6511/15/43/prepub

## Supplementary Material

Additional file 1 Arrive guidelines followed in the current study. Click here for file
