# Supplementary material for: A novel 11β-hydroxysteroid dehydrogenase type1 inhibitor CNX-010-49 improves hyperglycemia, lipid profile and reduces body weight in diet induced obese C57B6/J mice with a potential to provide cardio protective benefits
Source: BMC Pharmacol Toxicol. 2014 Aug 7;15:43. doi: 10.1186/2050-6511-15-43 (PMC4127523; doi:10.1186/2050-6511-15-43)
Supplement: Additional file 1 — Arrive guidelines followed in the current study. [file 2050-6511-15-43-S1.doc]

| **Additional File 1; ARRIVE GUIDELINES FOLLOWED IN THE CURRENT STUDY** | | | | | |  |
| --- | --- | --- | --- | --- | --- | --- |
| **Item** | | **Section** | **Recommendation** | **Remarks** | |  |
| **1** | | **TITLE** | **Provide as accurate and concise a description of the content of the article as possible.** | **Followed** | |  |
| **2** | | **ABSTRACT** | **Provide an accurate summary of the background, research objectives (including details of the species or**  **strain of animal used), key methods, principal findings, and conclusions of the study** | **Followed** | |  |
| **INTRODUCTION** | | | | | |  |
| **3** | | **Background** | **a. Include sufficient scientific background (including relevant references to previous work) to understand the motivation and context for the study, and explain the experimental approach and rationale.**  **b. Explain how and why the animal species and model being used can address the scientific objectives and, where appropriate, the study’s relevance to human biology** | **Provided**  **Provided some references to the similar models and the study outcomes (ref 14-20)** | |  |
| **4** | | **Objectives** | **Clearly describe the primary and any secondary objectives of the study, or specific hypotheses being tested.** | **Provided**  **(Page number 7, paragraph 3)** | |  |
| **METHODS** | | | | | |  |
| **5** | | **Ethical statement** | **Indicate the nature of the ethical review permissions, relevant licences (e.g. Animal [Scientific Procedures] Act 1986), and national or institutional guidelines for the care and use of animals, that cover the** **research.** | | **Mentioned under methods**  **(Page number 11, paragraph 1)** |  |
| **6** | | **Study design** | **For each experiment, give brief details of the study design, including:**  **a. The number of experimental and control groups.**  **b. Any steps taken to minimise the effects of subjective bias when allocating animals to treatment (e.g.randomisation procedure) and when assessing results (e.g., if done, describe who was blinded and when).**  **c. The experimental unit (e.g. a single animal, group, or cage of animals).** | | **The details are provided under –**  ***In vivo* efficacy studies in C57BL/6j mice on high fat diet** |  |
| **7** | **Experimental procedures** | | **For each experiment and each experimental group, including controls, provide precise details of all procedures carried out. For example:**  **a. How (e.g., drug formulation and dose, site and route of administration, anaesthesia and analgesia used [including monitoring], surgical procedure, method of euthanasia). Provide details of any specialist equipment used, including supplier(s).**  **b. When (e.g., time of day).** | | **Provided under different methods heading** | |
| **8** | **Experimental animals** | | **a. Provide details of the animals used, including species, strain, sex, developmental stage (e.g., mean or median age plus age range), and weight (e.g., mean or median weight plus weight range).**  **b. Provide further relevant information such as the source of animals, international strain nomenclature, genetic modification status (e.g. knock-out or transgenic), genotype, health/immune status, drug- or testnaı**  **¨ve, previous procedures, etc.** | | **The relevant information was given under methods section-**  ***In vivo* efficacy studies in C57BL/6j mice on high fat diet** | |
| **9** | **Sample size** | | **a. Specify the total number of animals used in each experiment and the number of animals in each experimental group.**  **b. Indicate the number of independent replications of each experiment, if relevant.** | | **Provided** | |
| **10** | **Allocating animals to**  **experimental groups** | | **Give full details of how animals were allocated to experimental groups, including randomisation or matching if done.** | | **Provided** | |
| **11** | **Experimental outcomes** | | **Clearly define the primary and secondary experimental outcomes assessed (e.g., cell death, molecular markers).** | | **Provided** | |
| **12** | **Statistical methods** | | **a. Provide details of the statistical methods used for each analysis.**  **b. Specify the unit of analysis for each dataset (e.g. single animal, group of animals).** | | **Provided** | |
| **RESULTS** | | | | | | |
| **13** | **Baseline data** | | **For each experimental group, report relevant characteristics and health status of animals (e.g., weight) before treatment or testing** | | **Provided wherever it is required (day 0 levels in the respective measurement graphs.**  **Eg. Fig 5A. 7A and 8A)** | |
| **14** | **Numbers analyzed** | | **Report the number of animals in each group included in each analysis. Report absolute numbers (e.g. 10/20, not 50%a).** | | **Provided under Fig legend** | |
| **15** | **Outcomes and estimation** | | **Report the results for each analysis carried out, with a measure of precision (e.g., standard error or confidence interval).** | | **Provided** | |
| **DISCUSSION** | | | | | | |
| **16** | **Interpretation/scientific implications** | | **a. Interpret the results, taking into account the study objectives and hypotheses, current theory, and other relevant studies in the literature.** | | **Discussed in detail** | |
| **17** | **Generalisability/translation** | | **Comment on whether, and how, the findings of this study are likely to translate to other species or systems, including any relevance to human biology.** | | **Briefly mentioned** | |
| **18** | **Funding** | | **List all funding sources (including grant number) and the role of the funder(s) in the study.** | | **Mentioned under**  **Acknowledgements** | |
